# Supplementary material for: Knowledge, attitudes, and practices related to COVID-19 among patients attending public dental clinics in Tanzania: A cross-sectional study
Source: PLoS One. 2022 Oct 27;17(10):e0276620. doi: 10.1371/journal.pone.0276620 (PMC9612478; doi:10.1371/journal.pone.0276620)
Supplement: S1 File — (DOCX) [file pone.0276620.s001.docx]

**Appendix IV – DODOSO (SWAHILI)**

**NAMBA YA** **DODOSO: ………….**

**MAELEKEZO**

Zungushia jibu lililo sahihi.

**SEHEMU I: TAARIFA ZA KIJAMII NA UCHUMI**

1. Umri (miaka): ......................

2. Jinsia:

a) Me b) Ke

3. Kiwango cha elimu:

a) Sijasoma b) Elimu ya msingi c) Elimu ya sekondari d) Chuo

4. Hali ya ndoa:

a) Sijaoa/Sijaolewa b) Nimeoa/Nimeolewa c) Naishi na bwana/bibi

d) Nimeachika e) Mjane/Mgane

5. Mahali/Eneo unapoishi: ...............................................

6. Kazi:

a) Mtumishi wa serikali b) Sekta binafsi c) Mjasiriamali d) Mkulima

e) Mwanafunzi f) Sina kazi e) Mengineyo………………………

**SEHEMU II: UELEWA JUU YA UVIKO 19**

7. Zifualtazo ni njia kuu za maambukizi ya Ugonjwa wa UVIKO 19

a) Vitonetone wakati wa kupiga chafya i. Ndiyo ii. Hapana

b) Njia isiyo ya moja kwa moja (bila kugusana) i. Ndiyo ii. Hapana

c) Maji maji ya mwili i. Ndiyo ii. Hapana

d) Vinyunyuzio wakati wa kupiga chafya i. Ndiyo ii. Hapana

e) Kujamiana i. Ndiyo ii. Hapana

8. Zifuatazo ni dalili kuu za UVIKO 19?

a) Homa i. Ndiyo ii. Hapana

b) Kuchoka mwili i. Ndiyo ii. Hapana

c) Miguu kuvimba i. Ndiyo ii. Hapana

d) Kichwa kuuma i. Ndiyo ii. Hapana

e) Nywele za mwili kunyonyoka i. Ndiyo ii. Hapana

f) Kupoteza uwezo wa kunusa i. Ndiyo ii. Hapana

g) Kutapika i. Ndiyo ii. Hapana

h) Mafua i. Ndiyo ii. Hapana

i) Kupumua kwa shida i. Ndiyo ii. Hapana

j) Kutokwa na damu puani i. Ndiyo ii. Hapana

k) Kikohozi kikavu i. Ndiyo ii. Hapana

l) Kuharisha i. Ndiyo ii. Hapana

9. Mtu aliyeambukizwa UVIKO 19 anaweza asioneshe dalili za ugonjwa. i. Ndiyo ii. Hapana

10. Zifuatazo ni njia za kujikinga dhidi ya ugonjwa wa UVIKO 19.

a) Kunawa mikono kwa maji tiririka na sabuni i. Ndiyo ii. Hapana

b) Kutumia vitakasa mikono i. Ndiyo ii. Hapana

c) Kushika macho, pua na mdomo mara kwa mara i. Ndiyo ii. Hapana

d) Kuvaa barakoa kwenye mikusanyiko i. Ndiyo ii. Hapana

e) Kukaa mbali mbali kwenye mikusanyiko i. Ndiyo ii. Hapana

f) Kuoga mara mbili kwa siku i. Ndiyo ii. Hapana

**SEHEMU III: MTAZAMO DHIDI YA UVIKO 19**

11. Je unadhani ugonjwa wa UVIKO 19 ni hataki kwa maisha?

i. Ndiyo ii. Hapana

12. Je unadhani tukifuata njia zilizoainishwa na Shirika la Afya la Dunia kutaweza zuia kuenea kwa ugonjwa wa UVIKO 19?

i. Ndiyo ii. Hapana

13. Je unadhani ni muhimu kwa wagonjwa kuchanja chanjo dhidi ya UVIKO 19?

i. Ndiyo ii. Hapana

14. Je unadhani kunaumuhimu was kupima vipimo vya kawaida vya kutambua UVIKO 19 (mfano.joto la mwili) wakati wa kuhudhuria kliniki ya meno?

i. Ndiyo ii. Hapana

15. Je unakubaliana kuwa, kujikinga mwenyewe dhidi ya UVIKO 19 kunasaidia kuwakinga wengine??

i. Ndiyo ii. Hapana

16. Je unadhani kuwa elimu ya afya juu UVIKO 19 inaumuhimu wowote?

i. Ndiyo ii. Hapana

**SEHEMU III: NJIA ZA KUJIKINGA DHIDI YA UVIKO 19**

17. Je ndani ya wiki moja umetumia njia zifuatazo kujilinda dhidi ya UVIKO 19?

a) Kuosha mikono kwa maji tiririka na sabuni i. Ndiyo ii. Hapana

b) Kutumia vitakasa mikono i. Ndiyo ii. Hapana

c) Kuvaa barakoa kwenye mikusanyiko i. Ndiyo ii. Hapana

d) Kutumia dawa za antibiotiki i. Ndiyo ii. Hapana

e) Kuoga kabla ya kuungana na wanafamilia pindi tu, nitokapo kazini au kwenye mizunguko yangu ya siku. i. Ndiyo ii. Hapana

f) Nimekula matunda jamii ya machungwa, tangawizi pamoja na vitamini c kwa ajili ya kuongeza uwezo wa kinga za mwili kupambana dhidi ya maradhi i. Ndiyo ii. Hapana

g) Kutumia dawa za mitishamba i. Ndiyo ii. Hapana

h) Nimetumia kitambaa kufunika mdomo wakati wa kukohoa na kupiga chafya i. Ndiyo ii. Hapana

i) Kufanya mazoezi ya mwili i. Ndiyo ii. Hapana

j) Nimesalimiana kwa kupeana mikono i. Ndiyo ii. Hapana

k) Kugusa macho, pua na mdomo kabla ya kunawa mikono. i. Ndiyo ii. Hapana
