# Supplementary material for: Knowledge, attitudes, and practices related to COVID-19 among patients attending public dental clinics in Tanzania: A cross-sectional study
Source: PLoS One. 2022 Oct 27;17(10):e0276620. doi: 10.1371/journal.pone.0276620 (PMC9612478; doi:10.1371/journal.pone.0276620)
Supplement: S2 File — (DOCX) [file pone.0276620.s002.docx]

**Appendix I – Questionnaire (English)**

**SERIAL NO; ………….**

**SECTION I: SOCIODEMOGRAPHIC CHARACTERISTICS**

1. Age: ............................. years.

2. Gender:

a) Male b) Female.

3. Education level

a) Informal education b) Primary c) Secondary d) Tertiary.

4. Marital status:

a) Single b) Married c) Cohabiting d) Divorced e) Widowed.

5. Residence: …………........................................

6. Occupation:

a) Civil servant b) Private sector c) Vendor d) Peasant e) Student f) Unemployed g) Others………………………………..

**SECTION II: KNOWLEDGE REGARDING COVID-19**

7. The following are the common modes of COVID-19 transmission?

a) Air droplets i. Agree ii. Disagree

b) Indirect contact i. Agree ii. Disagree

c) Body fluids i. Agree ii. Disagree

d) Aerosols i. Agree ii. Disagree

e) Sexual i. Agree ii. Disagree

8. The following are common clinical symptoms of COVID-19?

a) Fever i. Agree ii. Disagree

b) Fatigue i. Agree ii. Disagree

c) Limb edema i. Agree ii. Disagree

d) Headache i. Agree ii. Disagree

e) Alopecia i. Agree ii. Disagree

f) Loss of smell and taste i. Agree ii. Disagree

g) Vomiting i. Agree ii. Disagree

h) Flu i. Agree ii. Disagree

i) Difficulty in breathing i. Agree ii. Disagree

j) Nasal bleeding i. Agree ii. Disagree

k) Dry cough i. Agree ii. Disagree

l) Diarrhea i. Agree ii. Disagree

9. Person infected with COVID-19 can remain asymptomatic. i. Agree ii. Disagree

10. The following are preventive measures related to COVID-19.

a) Proper hand wash with soap and running water. i. Agree ii. Disagree

b) The use of sanitizer. i. Agree ii. Disagree

c) Frequently touching eyes, nose and mouth i. Agree ii. Disagree

d) Wearing masks in public places i. Agree ii. Disagree

e) Maintaining social distance i. Agree ii. Disagree

f) Taking bath twice a day i. Agree ii. Disagree

**SECTION III: ATTITUDES RELATED TO COVID 19**

11. Do you think COVID-19 is a life-threatening condition??

i. Yes ii. No

12. Do you think complying with the precaution measures introduced by the World Health Organization will prevent the spread of COVID-19?

i. Yes ii. No

13. Do you think it is important for people to be vaccinated against COVID-19?

i. Yes ii. No

14. Do you think that it is necessary to have a general screening for COVID-19 (e.g.measuring body temperature) during a regular dental checkup?

i. Yes ii. No

15. Do you agree that self-protection against COVID-19 is necessary to protect others?

i. Yes ii. No

16. Do you think that health education can play an important role to control COVID-19?

i. Yes ii. No

**SECTION IV: PRACTICE MEASURES RELATED TO COVID 19**

17. In the recent week have you practiced the following measures to protect yourself from COVID-19?

a) Washing hands with tapping water and soap i. Yes ii. No

b) Using hand sanitizer i. Yes ii. No

c) Wearing masks in public places i. Yes ii. No

d) Using antibiotics i. Yes ii. No

e) Showering immediately on getting home from work. i. Yes ii. No

f) Eating citrus fruits, ginger, and vitamin c for boosting immunity i. Yes ii. No

g) Using herbal remedies i. Yes ii. No

h) Using a handkerchief during coughing and sneezing i. Yes ii. No

i) Doing regular physical activity i. Yes ii. No

j) Shaking hands with others i. Yes ii. No

k) Touching eye, nose, and mouth before washing hands. i. Yes ii. No
