# Supplementary material for: Knowledge, attitudes, and practices related to COVID-19 among patients attending public dental clinics in Tanzania: A cross-sectional study
Source: PLoS One. 2022 Oct 27;17(10):e0276620. doi: 10.1371/journal.pone.0276620 (PMC9612478; doi:10.1371/journal.pone.0276620)
Supplement: Attachment — Submitted filename: response to reviewer comments and Cover letter.docx [file pone.0276620.s003.docx]

**The Academic Editor**	12^th^ September 2022

**PLOS ONE**

Sir

**RE: REVIEW OF MANUSCRIPT**

The above heading concerns.

We are very grateful to the entire Editorial Team of the journal and to the reviewers for the hard work you have done to ensure our manuscript entitled “Knowledge, attitudes, and practices related to COVID-19 among patients attending public dental clinics in Tanzania: A cross-sectional study

” is of high quality.

We have read and taken into consideration the constructive comments of both the academic editor and the reviewers. We have made all necessary amendments to our work. Track changes have been instituted in the manuscript in response to the editor and the reviewers’ comments.

The response is attached in the pages below as well.

Sincerely,

**Dr. Karpal Singh Sohal**

**The Corresponding Author**

**Comments:**

| **Academic Editor** | | |
| --- | --- | --- |
| **Editor’s comment** | **Authors response** | **Changes made on the page** |
| Please ensure that your manuscript meets PLOS ONE's style requirements, including those for file naming: | Thank you for the comment | Title page and the entire manuscript where appropriate |
| Please amend your current ethics statement to address the following concerns: a) Did participants provide their written or verbal informed consent to participate in this study?  b) If consent was verbal, please explain i) why written consent was not obtained, ii) how you documented participant consent, and iii) whether the ethics committees/IRB approved this consent procedure | We are humbled by the observation. We have included the statement to address that written consent was provided by the participants | Page 7; Line 137-138. |
| Thank you for providing the English version of the questionnaire used in your study. Please also include the Swahili version of the questionnaire as a supplementary file | We apologize for not including the Swahili version. We have included the Swahili version as a supplementary file. |  |
| Please review your reference list to ensure that it is complete and correct. If you have cited papers that have been retracted, please include the rationale for doing so in the manuscript text, or remove these references and replace them with relevant current references. Any changes to the reference list should be mentioned in the rebuttal letter that accompanies your revised manuscript. If you need to cite a retracted article, indicate the article’s retracted status in the References list and also include a citation and full reference for the retraction notice | We have reviewed the references for their completeness and correctness. None of the paper has been retracted. |  |
| Additional Editor Comments: Most of your references do not have journal issue number; where appropriate, they must be included. Also, ensure that you strictly follow the manuscript preparation guidelines of PLOS ONE--this is one of the conditions you must meet before your manuscript can be considered acceptable for publication | We appreciate the comment. Sir, we use Mendeley for citation, and in it, we used the PLOS ONE reference style. And it does not allow the issue to be displayed. We tried various PLOS journal styles present but non does display the issue. | None |
|  |  |  |
|  |  |  |
| **Reviewers** | | |
| **Reviewer 1** | | |
| **Reviewer’s comment** | **Authors response** | **Changes made on the page** |
| The article is well laid out and gives a great insight into the subject matter in Tanzania. | We are humbled by these comments from the reviewer. Thank you for the kind comments | None |
| Table 2 has disagree as the preferred option for body fluid and indirect contact as mode of transmissions, I think this should be reviewed | Comment appreciated. The ***disagree*** is the correct answer, as the question asked was the ‘common transmission mode’ had it been just “transmission mode” then ***agree*** would have been correct | None |
| Line 230 does not convey a clear idea | Thank you for the comment. The last sentences were indeed confusing, thus they have been deleted. | Page 16; Line 273. |
| Line 231 the discussion here has to be worked on. the author submission insinuates that the vaccine should be discouraged. I believe this is not the intent. | Thank you for pointing this out. We have changed the wording to show that it was an encouraging finding. | Page 16; Line 274. |
| Line 244, the author should elucidate more on the risks in the elderlies and how to mitigate these risks. | We appreciate the generous comment aimed at improving our work. We have acted upon the suggestion and did what the reviewer had suggested. | Page 17; Lines 294-295 |
| Line 255, dental is not necessary and should be deleted. | Suggestion well taken. The word dental has been deleted | Page 17; Line 305 |
|  |  |  |
| **Reviewer 2** | | |
| **Reviewer’s comment** | **Authors response** | **Changes made on the page** |
| The paper is relevant and could be considered for publication. | Thank you for the encouraging and kind comment. | None |
| Authors should however, expatiate on the mode of administration of the questionnaires and the assessment of reliability as stated in the methodology. | The comment has been well appreciated. Information on the mode of administration of the questionnaire has been added in the methodology section | Page 6; Lines 139 - 143 |
| Authors also did not state if there was a follow-up visit and this needs to be clarified. | Thank you for the comment. There was no follow-up. A statement about it has been added. | Page 6; Line143 |
| Furthermore, authors should state clearly if there was a pilot survey | We appreciate the comment. A statement regarding the pilot study has been included. | Page 6; Lines 136 - 137 |
